# Renal Endometriosis in a Postmenopausal Female Mimics Renal Cell Carcinoma

**DOI:** 10.7759/cureus.41133

**Published:** 2023-06-29

**Authors:** Kevin D Healey, Andrew B Herson, Davong D Phrathep, Conli Schwarz, Carlos E Ramos, Ahmad O Rifai

**Affiliations:** 1 Urology, Lake Erie College of Osteopathic Medicine, Jacksonville, USA; 2 Urology, Advanced Urology Institute, Panama City, USA; 3 Department of Research and Publications, The Virtual Nephrologist, Lynn Haven, USA

**Keywords:** renal cell carcinoma (rcc), multicystic renal mass, recurrent uti, postmenopausal female, endometriosis

## Abstract

A 61-year-old postmenopausal female with a past medical history of type 2 diabetes, nephrolithiasis, and recurrent urinary tract infections presented to an outpatient urology clinic with a chief complaint of urinary frequency, urgency, and burning after micturition. Associated symptoms included nausea, a low-grade fever with chills, and right flank pain. After treatment with antibiotics did not relieve all of her symptoms, imaging was obtained, showing a cystic mass with calcifications in the right kidney. Following laparoscopic partial right nephrectomy and total hysterectomy with bilateral salpingo-oophorectomy, pathological examination of the right kidney mass highlighted endometrial stromal cells consistent with endometriosis of the right kidney. The left ovary also contained endometrial stromal cells, confirming another diagnosis of endometriosis of the left ovary. This case highlights the importance of considering renal endometriosis in the differential diagnosis of renal masses in women, even if they are postmenopausal.

## Introduction

Endometriosis is a condition of abnormal endometrial tissue growth and implantation outside of the uterus [1]. Typically, endometriosis occurs in women of reproductive age, and it represents the most common cause of pelvic pain in women [2]. Although patients can be asymptomatic, other symptoms of endometriosis include dysmenorrhea, menorrhagia, and dyspareunia [1]. Symptoms of endometriosis usually occur during menstruation because the abnormal endometrial tissue growth and implantation act like the endometrium, where it will thicken, break down, and bleed [3]. Extrapelvic endometriosis is uncommon, but current literature suggests the urologic organ distribution to be less than 5% when it involves the kidney [2]. In recent reports, endometriosis has been documented to occur in postmenopausal females and has shown the potential to implant in the kidneys. Renal endometriosis can mimic several urologic processes, including hematuria, flank pain, and ureteral obstruction [2]. In this report, we present a unique case of renal endometriosis causing flank pain and a cystic mass with calcification in a postmenopausal female.

## Case presentation

A 61-year-old female with a past medical history of type 2 diabetes, nephrolithiasis, and urinary tract infections (UTIs) presented to our outpatient urology clinic with complaints of a two-day history of urinary frequency, urgency, and burning at the end of micturition. She had been experiencing nausea and low-grade fever with chills. On physical examination, the patient displayed discomfort and right flank pain. Urinalysis was performed, showing minor terminal hematuria and leukocyturia. The patient was started on oral nitrofurantoin 100 mg twice per day for seven days.

**Figure 1:**
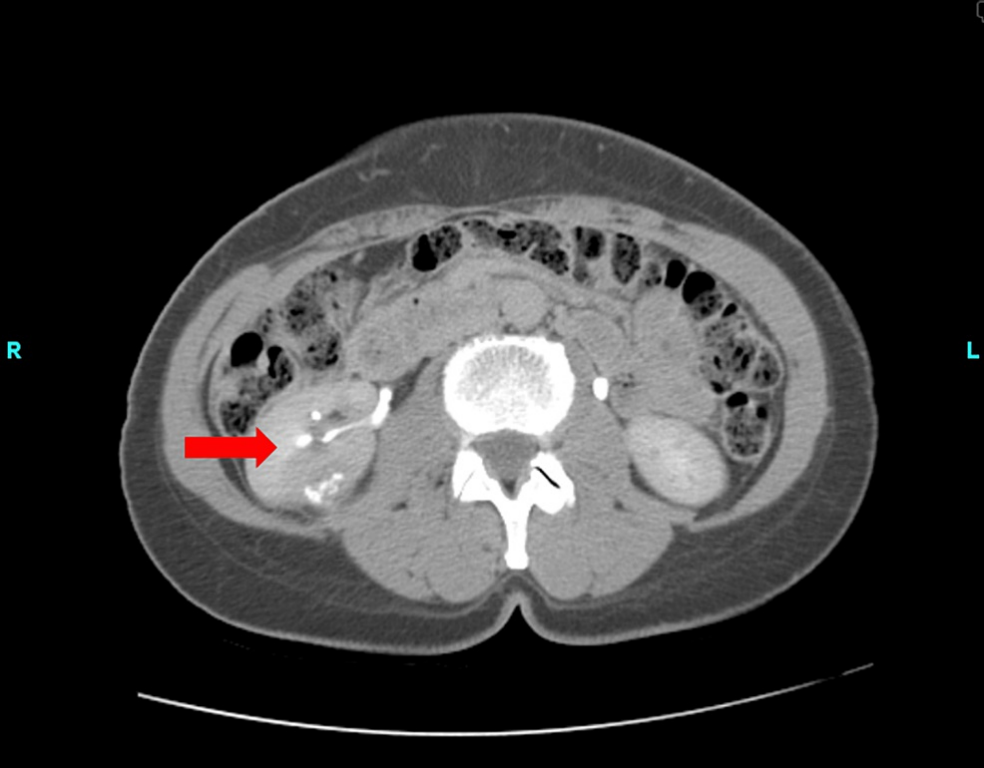
CT IV pyelogram CT IV pyelogram showing a complex cystic mass with calcifications of the right kidney (red arrow). CT IV: computed tomography intravenous

After completing the prescribed antibiotic course, her symptoms began to subside and showed a significant improvement in severity. However, despite the treatment, her flank pain remained present. As a result of her persistent symptoms, a computed tomography intravenous pyelography (CT IVP) and a CT scan of the abdomen and pelvis without contrast were ordered to gain a more comprehensive understanding of the underlying cause. The CT IVP demonstrated a mildly dilated right ureter with no evidence of obstruction. However, there was a complex cystic mass with calcifications posteriorly in the midportion of the kidney (Figure 1).

The central cystic part had 17 Hounsfield units. The heavily calcified wall was 35 Hounsfield units, enhanced to 89 Hounsfield units on post-imaging. This was further classified as a Bosniak 3 that measured 3 cm in diameter. The CT scan without contrast of the abdomen and pelvis revealed bilateral ovarian cysts, with a dominant left ovarian cyst measuring 3.7 cm. At this point in time, the patient agreed to a laparoscopic partial right nephrectomy.

The renal mass was not immediately visible, and further exploration was required to locate it. The mass was located using intraoperative ultrasound, and it was carefully dissected from lateral to medial and upper to lower pole until it was completely excised. Fluid within the mass was noted to be straw-colored. Endometriosis was diagnosed, and a subsequent total hysterectomy with bilateral salpingo-oophorectomy was performed. The surgical procedures were both successful and well tolerated by the patient, with no complications.

The right renal mass, uterus, fallopian tubes, and ovaries were sent to pathology. Immunoperoxidase stain of the right kidney mass with the appropriate control highlights endometrial stromal cells consistent with endometriosis of the right kidney. Additionally, the left ovary also contained endometrial stromal cells, confirming another diagnosis of endometriosis of the left ovary.

## Discussion

Endometrial tissue changes throughout a woman’s menstrual cycle and is shed, marking the onset of menstruation. When the endometrial tissue is present outside of the uterus, it is referred to as endometriosis [1]. Endometriosis most commonly occurs in the ovaries, fallopian tubes, and vagina [4]. This further complicates the diagnosis of endometriosis of the kidney, as seen in our case. Endometriosis occurring at distant sites from gynecological organs is rare and has only been documented sparsely [2]. While the cause of endometriosis is uncertain, retrograde menstruation is postulated as a potential cause [1]. At 61 years of age, our patient is postmenopausal, suggesting an alternative cause for her abnormal case of renal endometriosis. The patient's postmenopausal hematuria also raises concern for a potential urological malignancy.

Most commonly occurring during a woman's fertile years, endometriosis can present with painful menses (cramping and heavy bleeding), precoital and postcoital pain, and pain with micturition and defecation. Endometriosis can also present with intermenstrual bleeding [1]. The patient presented to the office with right flank pain, urinary urgency, and burning upon micturition. All of these symptoms, along with her medical history of nephrolithiasis and recurrent UTIs, pointed to a UTI. After being treated, some of her symptoms persisted. Because the patient is in a postmenopausal state, the typical symptoms of endometriosis are absent. This complicated the patient’s diagnosis. Badri et al. describe an abnormal case of renal endometriosis presenting in a 45-year-old premenopausal woman; Yang et al. also describe a similar case presenting in a 48-year-old premenopausal woman; and Yang et al. report a 37-year-old premenopausal woman with renal endometriosis misdiagnosed as a renal tumor [2,4,5]. While similarities are seen in all cases, the major difference lies in the patients’ ages. Symptoms that occur with the timing of the menstrual cycle can help with the diagnosis. In our case, since there is no menstrual cycle to use as a reference for the timing of symptoms, it can be easy to misdiagnose. The treatment of pain also differs. In cases of premenopausal endometriosis, oral contraceptives can help alleviate pain by creating a hypoestrogenic state. While confirmatory diagnosis depends on histopathological examination for renal endometriosis, if hematuria and flank pain are diminished following medication, this may help in the diagnosis of renal endometriosis [5]. However, since most cases of renal endometriosis are rare and present in an atypical fashion, they are usually diagnosed postoperatively.

## Conclusions

Due to the patient’s age, post-menopausal state, and the unusual, rare location of the endometriosis, the right kidney, an embryonic theory of endometriosis comes into question as a potential cause of this patient's renal endometriosis. The differential diagnosis for a cystic renal mass seen on imaging should include renal endometriosis, regardless of the patient’s age. However, malignancies and other serious diagnoses like hemorrhagic cysts should remain at the top of the differential diagnosis list, as renal endometriosis is rare even though it can mimic a more serious cause of renal masses, such as renal cell carcinoma.
